# The LifeLines Cohort Study: a resource providing new opportunities for environmental epidemiology

**DOI:** 10.1186/s13690-016-0144-x

**Published:** 2016-08-01

**Authors:** Wilma L. Zijlema, Nynke Smidt, Bart Klijs, David W. Morley, John Gulliver, Kees de Hoogh, Salome Scholtens, Judith G. M. Rosmalen, Ronald P. Stolk

**Affiliations:** 1 Department of Epidemiology, University Medical Center Groningen, HPC CC72, University of Groningen, PO Box 30.001, 9700 RB Groningen, The Netherlands; 2 Department of Epidemiology and Biostatistics, MRC-PHE Centre for Environment and Health, Imperial College London, London, UK; 3 Swiss Tropical and Public Health Institute, Basel, Switzerland; 4 University of Basel, Basel, Switzerland; 5 LifeLines Cohort Study, Groningen, The Netherlands; 6 Department of Psychiatry, University Medical Center Groningen, University of Groningen, Groningen, The Netherlands

**Keywords:** Prospective cohort study, Environmental exposures, Harmonization, Chronic disease

## Abstract

**Background:**

Lifelines is a prospective population-based cohort study investigating the biological, behavioral and environmental determinants of healthy ageing among 167,729 participants from the North East region of the Netherlands. The collection and geocoding of (history of) home and work addresses allows linkage of individual-level health data to detailed exposure data. We describe the reasons for choosing particular assessments of environmental exposures in LifeLines and consider the implications for future investigations.

**Methods:**

Exposure to ambient air pollution and road traffic noise was estimated using harmonized models. Data on noise annoyance, perceived exposure to electromagnetic fields, perceived living environment, and neighborhood characteristics were collected with questionnaires. A comprehensive medical assessment and questionnaires were completed in order to assess determinants of health and well-being. Blood and urine samples were collected from all participants and genome wide association data are available for a subsample of 15,638 participants.

**Results:**

Mean age was 45 years (standard deviation (SD) 13 years), and 59 % were female. Median levels of NO_2_ and PM_10_ were 15.7 (interquartile range (IQR) 4.9) μg/m^3^ and 24.0 (IQR 0.6) μg/m^3^ respectively. Median levels of daytime road traffic noise were 54.0 (IQR 4.2) dB(A).

**Conclusions:**

The combination of harmonized environmental exposures and extensive assessment of health outcomes in LifeLines offers great opportunities for environmental epidemiology. LifeLines aims to be a resource for the international scientific community.

## Background

Environmental epidemiology has contributed to public health by focusing on the reduction of harmful exposures from the environment. Smoke-free legislation [1], control of particulate air pollution [2], and the introduction of proper sanitation [3] are examples of policies that resulted in substantial benefits for public health. Current efforts in the Netherlands to reduce exposure to air pollution include the introduction of “environmental zones” where only clean vehicles are allowed, the mandatory use of particulate filters in vehicles, and the introduction of clean public transport. Although large efforts have been made, more is to gain in environmental epidemiology, both in terms of public health policy and research. Prevention of harmful environmental exposures is important because many of these exposures are often involuntary and outside the immediate control of the individual, and many individuals may be affected by the same pollution source.

Studying health effects of environmental exposures can be challenging. Environmental exposures often occur in low concentrations and in complex mixtures. Most health outcomes of interest have many underlying risk factors whose effects may be much stronger than those of the environmental exposure. Risks associated with environmental exposures are generally small [4], making them difficult to detect. Although effect sizes are small, impacts on health are still substantial because of the large number of exposed persons. Population based cohort studies with large sample sizes are needed to investigate the complex interaction between genetic, behavioral, and environmental factors in the development of multifactorial chronic diseases. Collaboration between cohort studies could help understand environmental causes of disease, and the advantages of such an approach are widespread. Such collaboration will lead to large sample sizes and wide exposure ranges. Furthermore, results can be compared across regions, in which not only the exposures, but also genetic, social and cultural factors vary, making the generalizability of study results larger. Generalizing study results across large regions in Europe is important for establishing European exposure norms and guidelines. The harmonization and standardization of population studies will enable the assembling of data in valid and effective ways. To this end, LifeLines aims to implement standardized and harmonized data collection methods.

LifeLines is one of eight studies participating in the BioSHaRE (Biobank Standardisation and Harmonisation for Research Excellence in the European Union, www.bioshare.eu) project. Within the framework of this European harmonization initiative, tools were developed for data harmonization, database integration and federated data analyses [5]. Standardized and validated methods were used wherever possible, and a part of the dataset (e.g., educational level, alcohol and tobacco use) has been retrospectively harmonized with other European cohorts [6].

Our aim is to inform researchers on the choice of data collection methods and methodology of data on environmental exposures in LifeLines. Descriptive information of selected exposure and cohort data will be reported. By describing these features of LifeLines we aim to highlight methodological choices made in the context of large population based cohort studies exposed to multiple environmental stressors.

## Methods

### Study design and participants

LifeLines is a multi-disciplinary prospective population based cohort study examining the health and health-related behaviors of persons living in the North East region of the Netherlands. The cohort was established to facilitate research on complex interactions between environmental, phenotypic and genetic factors in the development of chronic diseases [7, 8]. Recruitment of study participants took place between 2006 and 2013. In the Netherlands, all inhabitants are registered with a general practitioner. A large number of general practitioners from the Northern provinces of the Netherlands participated in the recruitment and invited all their patients between ages 25 and 50 years. Individuals who agreed to participate were asked to indicate whether their family members would also be willing to participate. In addition, individuals could also register themselves via the LifeLines website. The sample was recruited from the three Northern provinces of the Netherlands, but this was not a requirement for inclusion of family members [8]. Characteristics of adult LifeLines participants are broadly representative for the adult population of the North of the Netherlands [9].

Baseline data were collected from 167,729 participants, aged 6 months to 93 years. Follow-up is planned for at least 30 years, with questionnaires administered every 1.5 years, and a renewed physical examination scheduled every five years. Participants visited one of the LifeLines research sites for a physical examination, including spirometry, electrocardiogram (ECG), blood pressure measurements, anthropometry, cognition tests, and a psychiatric interview. Fasting blood and 24-h urine samples were collected from all participants, and genome wide association data are currently available for a subsample of 15,638 participants. An extensive baseline questionnaire was completed at home, including questions on history of illness, health related quality of life, lifestyle, socioeconomic status, psychosocial stress, work (profession, working hours), psychosocial characteristics, and medication use. Linkage will be established with records from general practitioners and health registries. A comprehensive and detailed overview of the available data is presented in the online LifeLines Data Catalogue (www.lifelines.net).

### Geocoding

All participants’ home addresses were geocoded. In addition to the most recent home address, address history is available from the Municipal Personal Record Database. This governmental registry contains personal data of all individuals who live or have lived in the Netherlands. Data on address history provide insights in residential mobility and length of exposure to different environments, and allows assessment of long-term exposures relevant for life course epidemiology. Work addresses were also collected and will be geocoded as well, allowing for outdoor exposure estimation of air pollution and noise at both home and work location. Most studies estimate exposure to road traffic noise and air pollution at the home addresses of participants (e.g., [10, 11]), assuming this to be a good indicator of personal exposure. Individuals spend a large amount of their time at the work address, and combining exposures from both locations will result in better estimations of exposure [12]. A detailed description of the environmental exposures assessed in LifeLines is described below, and is summarized in Table 1.

**Table 1:** Overview of environmental exposures in the LifeLines Cohort Study

Ambient air pollution	
*ESCAPE models*	
NO_2_	Nitrogen dioxide
NO_2_ background	Background level of nitrogen dioxide
PM_2.5_	Particulate matter with diameter ≤2.5 μm
PM_2.5_ absorbance	Reflectance on PM_2.5_ filters, i.e., marker of black carbon
PM_10_	Particulate matter with diameter ≤10 μm
*EU-wide models*	
NO_2_	Nitrogen dioxide
PM_10_	Particulate matter with diameter ≤10 μm
Road traffic noise (CNOSSOS-EU model)	
L_day_	A-weighted equivalent noise level over the 12-hour day time period from 07.00 to 19.00 hour
L_evening_	A-weighted equivalent noise level over the 4-hour evening time period from 19.00 to 23.00 hours
L_night_	A-weighted equivalent noise level over the 8-hour night time period from 23.00 to 07.00 hours
L_aeq16_	A-weighted equivalent noise level over the 16-hour day and evening time period from 07.00 to 23.00 hours
L_den_	A-weighted equivalent noise level for the day-evening-night time period of 24 hours, with a 10 dB penalty added to the levels between 23.00-07.00 hours and a 5 dB added to the levels between 19.00-23.00 hours to reflect extra noise sensitivity during night and evening
L_aeq_ 0-23 hours	Hourly noise estimates
Questionnaire data	
Noise annoyance	
Perceived exposures to power lines, mobile phone masts	
Mobile phone use	
Exposure to secondhand smoke	
Perceived living environment	
Database linkage	
Neighborhood characteristics (Statistics Netherlands)	Neighborhood level demographic and socioeconomic figures
LISA employment register	Location, type of establishment, number of employees

*ESCAPE* European Study of Cohorts for Air Pollution Effects, *EU-wide* European-wide, *CNOSSOS-EU* Common Noise Assessment Methods in Europe

### Ambient air pollution

Exposure to air pollution has been related to various health outcomes, including respiratory diseases [13] and cardiovascular disease [14]. In LifeLines, exposure to ambient air pollution was estimated using land use regression (LUR) models developed for the European Study of Cohorts for Air Pollution Effects (ESCAPE) [15, 16] and using European wide LUR models enhanced with satellite derived air pollution estimates [17]. It was chosen to implement these air pollution models in LifeLines because both models are advantageous regarding comparability across studies in other European regions.

Within the ESCAPE project, LUR models for various European study areas were developed using a standardized approach. ESCAPE LUR models were developed for NO_2_ (nitrogen dioxide), NO_2_ background, PM_2.5_ (particulate matter with a diameter ≤2.5 μm), PM_2.5_ absorbance (reflectance on PM_2.5_ filters, i.e., a marker of black carbon), and PM_10_ (particulate matter with a diameter ≤10 μm), and were based on annual average concentrations from an intensive monitoring campaign and GIS (geographic information system) derived predictor variables (e.g., distance to the nearest major road, traffic intensity, built-up land, population density, altitude). LUR models were developed using measurements carried out in 2009–2010 and predictor variable data for the same years. Model performance was evaluated by leave-one-out cross validation. The adjusted explained variability in measured concentrations (R^2^) was 0.85 for NO_2_, 0.83 for NO_2_ background, 0.66 for PM_10_, 0.64 for PM_2.5_, and 0.91 for PM_2.5_ absorbance [15, 16].

In addition, exposure to ambient PM_10_ and NO_2_ is available from European(EU)-wide models. These EU-wide models incorporate GIS-derived land use, road network, and topographic data, as well as satellite-derived estimates of ground level concentrations for PM_2.5_ (as an indicator of PM_10_) and NO_2_. Model development follows the ESCAPE procedure to construct the multiple linear regression equations, and are applicable for years 2005, 2006 (NO_2_) and 2007 (NO_2_ and PM_10_). Models were evaluated against measured PM_10_ and NO_2_ concentrations at an independent subset of sites reserved for this purpose. The adjusted explained variability in measured concentrations (R^2^) was 0.48 − 0.58 for NO_2_, and 0.22 − 0.50 for PM_10_ [17].

The main difference between the ESCAPE and EU-wide models is that the ESCAPE models are region specific, while EU-wide models are developed for a much larger area. ESCAPE models are developed for 20 (PM) to 36 (NO_2_) European regions and EU-wide models for 17 countries in Western Europe. In addition, monitoring data used in ESCAPE models originated from a monitoring campaign specifically conducted for the ESCAPE-project with monitoring sites selected for this purpose, whereas monitoring data for the EU-wide models were obtained from regulatory monitoring networks. A study including Lifelines and other cohorts falling in ESCAPE study areas (e.g., the British cohort EPIC-Oxford, also involved in BioSHaRE) should use air pollution exposures estimates from the relevant ESCAPE models. However, the EU-wide model can also provide air pollution exposure estimates for areas falling outside the ESCAPE study areas, and will therefore allow research including Lifelines plus non-ESCAPE cohorts (e.g., the Norwegian cohort HUNT, also involved in BioSHaRE).

### Road traffic noise

Environmental noise has been related to a variety of adverse outcomes, including hearing loss, annoyance, sleep disturbance, cognitive impairment, and cardiovascular disease [18]. In LifeLines, road traffic noise was estimated using an implementation of the Common Noise Assessment Methods in Europe (CNOSSOS-EU) noise modeling framework [19], which was developed as a common methodology for noise modeling across Europe. This noise model was preferred since it allows comparison of results from different countries. The CNOSSOS-EU noise model implemented within LifeLines uses lower resolution source data because the highest resolution input data at national or large regional level is either unavailable, expensive or would be too computationally intensive to process. The performance of CNOSSSOS-EU using the lower resolution inputs has been shown to be reasonable for application in epidemiological studies. The model’s exposure ranking, i.e., prediction of noisier and quieter sites, was adequate (Spearman’s rank = 0.75; p <0.001), but the predicted noise levels have relatively large errors (root mean square error (RMSE) = 4.46 dB(A)) [20].

The CNOSSOS-EU framework contains empirically derived equations to determine the initial noise level based on traffic flow and sound attenuation (i.e., sound reduction or damping) based on known environmental factors and physical processes. To estimate source noise on road segments in the Netherlands, traffic information was obtained including hourly flow of passenger cars, heavy goods vehicles and their average speeds. As detailed land cover data were not available to allow positioning of the receptor at the most exposed façade, a coarser land cover data set was used to approximate urban fabric. The sound propagation model was based on the CORINE (Coordination of information on the environment) land cover dataset that has a European wide coverage accurate to 100 m for major land cover types [21]. In particular, the distinction between urban fabric and areas of vegetation was made. Traffic data originated from year 2009 and landcover data from 2006. Full details of this approach are described by Morley and colleagues (2015) who show that lower resolution data may be used within the CNOSSOS-EU noise model to obtain representative exposure estimates [20].

### Perceived exposures

Besides the actual exposure to environmental factors, perceived exposure and concerns about the health risks associated with the exposure might influence health outcomes [22]. When studying particular exposures, for example electromagnetic fields, the public’s perception of the health risks is as relevant to health as the exposure itself. Moreover, sometimes perceived exposures have stronger associations with diminished health than the actual exposures [23]. Noise annoyance from eight different sources was measured using a standardized self-report questionnaire. The sources of noise annoyance include for example air, road and rail traffic. This questionnaire originates from the International Organization for Standardization (ISO) guideline, which provides specifications for socio-acoustic surveys and social surveys that include questions on noise effects [24]. Similar noise annoyance questions were used in the HYENA study on HYpertension and Exposure to Noise near Airports [25]. Perceived exposure to electromagnetic fields is measured using a questionnaire on perceived exposure to power lines and mobile phone masts. Participants were asked to what extent they think they are exposed to radiation from power lines and mobile phone masts, and whether they perceive this as bad for their health (adapted from [26]). In addition, a number of questions on the use of mobile phones (e.g., average time per week using mobile phone; on which side of the head) were included. These questions were adapted from the UKBiobank questionnaire [27], which is one of the cohorts involved in the BioSHaRE project. Exposure to second-hand smoke is assessed with questions about the duration and place (household, workplace) of exposure, and originate from the European Community Respiratory Health Survey [28].

In addition to work addresses, information was collected on working hours and type of profession, which may be relevant for occupational exposure studies. In the next follow-up questionnaire, LifeLines will measure how participants perceive their living environment. The questionnaire comprises of nine items investigating characteristics of the physical and social living environment as perceived by the respondent (e.g., neighborhood satisfaction, social interaction with neighbors), and was based on the 2010 health survey of the Dutch Community Health Services (Dutch name: GGD Gezondheidsenquête 2010) [29] and the 2006 WoON questionnaire (Dutch name: WoonOnderzoek Nederland) [30].

### Neighborhood characteristics

Various neighborhood characteristics have been associated with health, ranging from cardiometabolic risk factors [31] to life expectancy [32]. Data on neighborhood characteristics from Statistics Netherlands are available for linkage to the LifeLines database. Statistics Netherlands publishes demographic and socioeconomic figures for municipalities, districts and neighborhoods [33]. These figures cover various themes, for example housing, education, income, and land use. Such information enables to investigate the impact of neighborhood conditions on health.

Furthermore, the LISA employment register (www.lisa.nl) is linked to the LifeLines database. This register contains nationwide information on locations (geocoded at the address level) of establishments where paid work is done. The database also contains information about the type of establishment (i.e., restaurants, hospitals, shops) and the number of employees. Using the LISA employment register, it is possible to investigate the density and distance to specific facilities in relation to various health outcomes.

### Data access and linkage

LifeLines has adopted an open protocol, meaning that within the standing infrastructure additional data and biomaterial collection, and linkage with other (environmental) data sources can be implemented, for example for the purpose of environmental biomonitoring of exposure and response. One example is LifeLines DEEP, an add-on study where in a subsample of participants additional biological samples (feces, exhaled air) were collected, additional blood analyses were undertaken, and additional questionnaires were filled out [34]. Other exposures types, such as domestic radon, electromagnetic fields, harmful chemicals (e.g., pesticides) are of interest for research in LifeLines, and proposals for additional environmental exposures assessments relevant for healthy ageing are warmly welcomed. Furthermore, biological samples are stored for future analyses, enabling for example measurement of exposure biomarkers. Data and biomaterials are provided on a fee-for-service basis and may be used for scientific research only. Public and private researchers, from inside and outside the Netherlands are invited to submit a research proposal to the LifeLines Research Office (LLscience@umcg.nl). Quality control of data is done by trained medical students and data managers, using Standard Operating Procedures (SOPs). Data is released within a remote system (LifeLines workspace) running on a high performance computer cluster, which ensures data quality and security. The LifeLines research website (www.lifelines.net) provides the details of the application process, the data collection, and an overview of publications with LifeLines data.

## Results

The first data release of the baseline sample consisted of 95,432 participants, of which 58.7 % were female. Mean age was 45.2 years (standard deviation (SD) 12.6 years), and men were more often current smokers than women (23.8 and 20.4 %, respectively). Overall, mean body mass index (BMI) was 26.1 kg/m^2^ (SD 4.3 kg/m^2^), and mean systolic blood pressure was 125.6 mmHg (SD 15.3 mmHg). Forced vital capacity (FVC), a measure for lung function assessed with spirometry, was on average 4.5 (SD 1.1) liters. Most participants live in rural (i.e., <500 addresses per km^2^) neighborhoods (41.2 %) (Table 2).

**Table 2:** Sample characteristics by sex of the LifeLines Cohort Study

	Women	Men	Total
N^a^ (%)	56 053 (58.7)	39 379 (41.3)	95 432
Age (years)	44.9 (12.6)	45.7 (12.7)	45.2 (12.6)
Education (%)			
No or primary	3.2	3.0	3.1
Lower or preparatory vocational	12.4	16.2	14.0
Lower general secondary	16.0	11.7	14.2
Intermediate vocational or apprenticeship	30.6	31.0	30.8
Higher general secondary or pre-university secondary	9.9	7.0	8.7
Higher vocational or university	28.1	31.0	29.3
Current smokers (%)	20.4	23.8	21.8
BMI (kg/m^2^)	25.9 (4.7)	26.4 (3.7)	26.1 (4.3)
SBP (mmHg)	122.1 (15.3)	130.7 (14.0)	125.6 (15.3)
DBP (mmHg)	71.8 (8.7)	76.6 (9.3)	73.8 (9.3)
FVC (L)	3.9 (0.6)	5.4 (0.9)	4.5 (1.1)
FEV1 (L)	3.0 (0.6)	4.1 (0.8)	3.5 (0.8)
Urbanity ^b^ (%)			
Rural	40.8	41.9	41.2
Semi-rural	24.3	25.4	24.8
Intermediate urban-rural	17.5	16.7	17.1
Semi-urban	10.6	10.1	10.4
Urban	6.8	6.0	6.5

Means (SD) are presented for continuous variables, and percentages are presented for categorical variables

^a^ Data are based on the first data release of *n* = 95,432

^b^Average number of addresses per km^2^ within a range of 1 kilometer, categorized into five levels ranging from rural (<500 addresses per km^2^) to urban (≥2500 addresses per km^2^)

*Abbreviations: BMI* body mass index, *SBP* systolic blood pressure, *DBP* diastolic blood pressure, *FVC* forced vital capacity, *FEV1* forced expiratory volume in 1 second

**Fig. 1:**
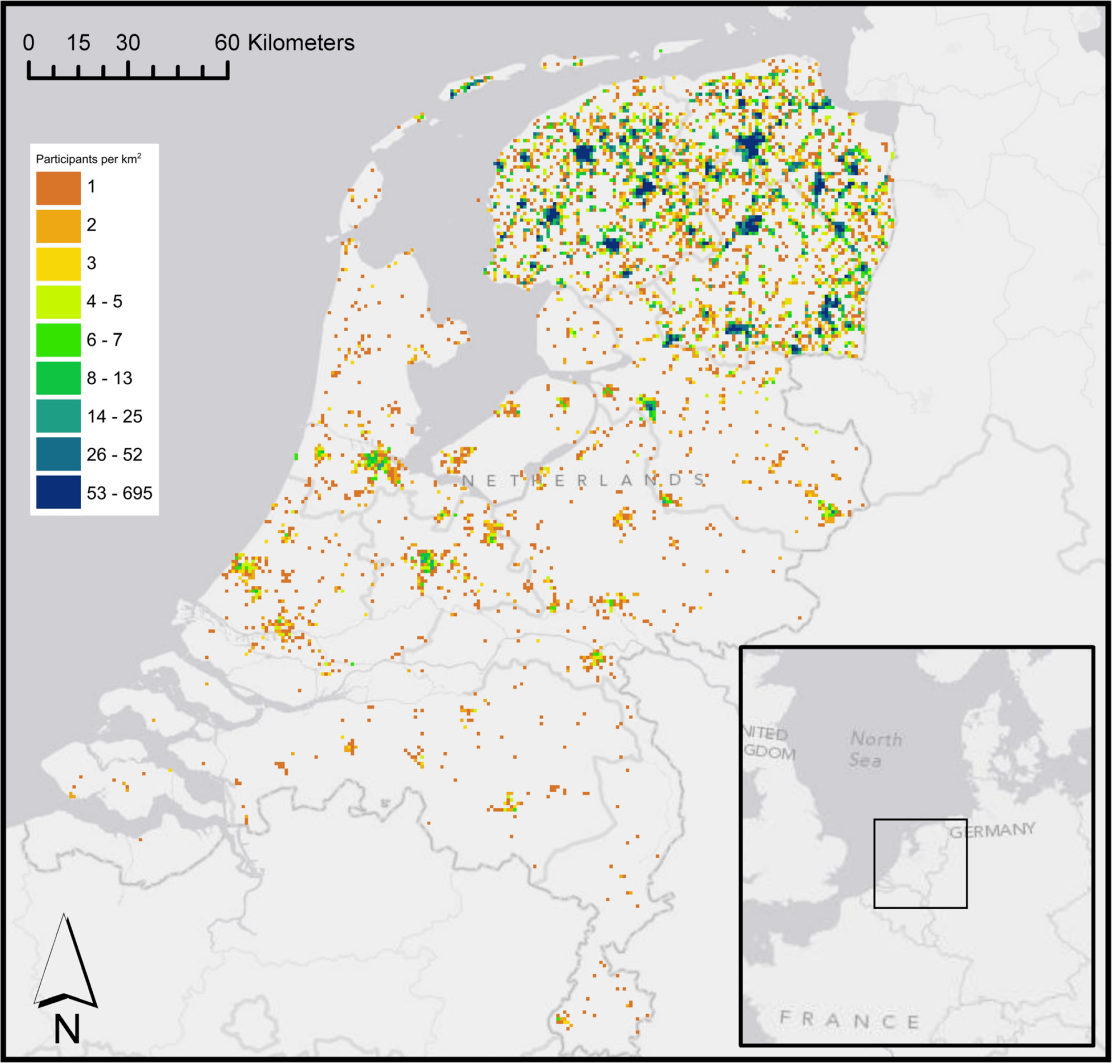
LifeLines study area and number of participants per square kilometer. Participants were aggregated and plotted at the center of each 1 km grid cell

**Fig. 2:**
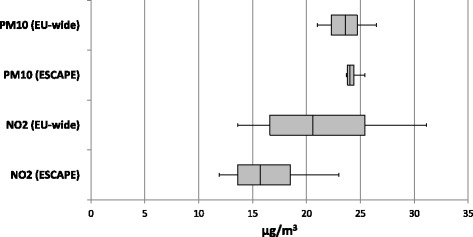
Distribution of nitrogen dioxide and particulate matter (≤10 μm) exposure in the LifeLines Cohort Study with estimates based on ESCAPE land use regression model and EU-wide land use regression model. Medians, 25^th^, and 75^th^ percentiles are shown in the box, whiskers indicate 5^th^ and 95^th^ percentiles. ESCAPE = European Study of Cohorts for Air Pollution Effects; EU-wide = European-wide

Most participants live in the three Northern provinces of the Netherlands, but part of the participants (approximately 3 %) live elsewhere in the country (Fig. 1). Median levels of NO_2_ were 15.7 (interquartile range (IQR) 4.9) μg/m^3^ (ESCAPE) and 20.6 (IQR 7.9) μg/m^3^ (EU-wide), and 24.0 (IQR 0.6) μg/m^3^ (ESCAPE) and 23.6 (IQR 2.4) μg/m^3^ (EU-wide) for PM_10_ (Fig. 2). Correlation between ESCAPE-LUR modeled NO_2_ and satellite-enhanced LUR modeled NO_2_ was high (Spearman’s rho = 0.86), while correlation for PM_10_ from both models was moderate (Spearman’s rho = 0.54).

**Fig. 3:**
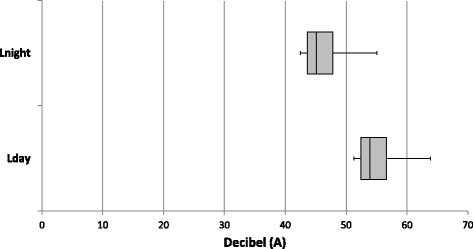
Distribution of road traffic noise exposure L_day_ and L_night_ in the LifeLines Cohort Study. Medians, 25^th^, and 75^th^ percentiles are shown in the box, whiskers indicate 5^th^ and 95^th^ percentiles. dB(A) = decibel (A); L_day_ = A-weighted equivalent noise level over the 12-h day time period from 07:00 to 19:00 h; L_night_ = A-weighted equivalent noise level over the 8-h night time period from 23:00 to 07:00 h

**Fig. 4:**
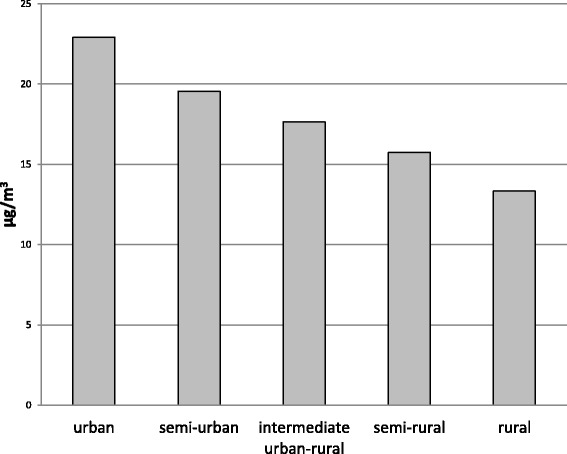
Median exposure to ambient nitrogen dioxide (NO_2_; based on ESCAPE model) according to degree of urbanity within the LifeLines Cohort Study

**Fig. 5:**
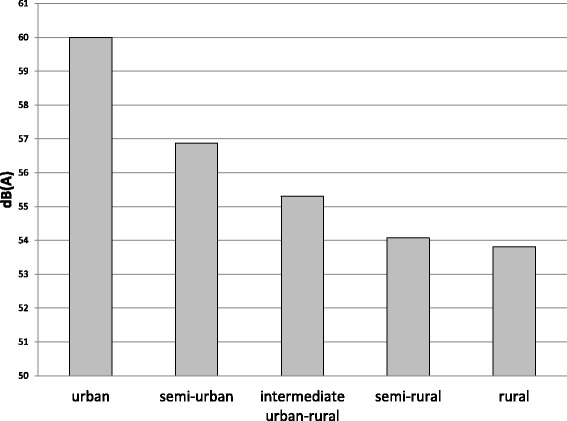
Median exposure to road traffic noise (L_den_; average A-weighted noise level estimated over a 24 h period) according to degree of urbanity within the LifeLines Cohort Study

Median levels of road traffic noise were 54.0 (IQR 4.2) dB(A) (decibel (A)) for L_day_ (A-weighted equivalent noise level over the 12-h day time period from 07.00 to 19.00 h) and 45.1 (IQR 4.2) dB(A) for L_night_ (A-weighted equivalent noise level over the 8-h night time period from 23.00 to 07.00 h) (Fig. 3). LifeLines participants living in urban neighborhoods had highest exposure to air pollution (NO_2_; Fig. 4) and 24-h road traffic noise (L_den_) (Fig. 5), compared to participants in neighborhoods of lower degree of urbanity. The correlation between (ESCAPE modeled) NO_2_ and urbanity was Spearman’s rho: 0.88 (p < 0.001) and Spearman’s rho: 0.42 (p < 0.001) for L_den_ and urbanity.

## Discussion

With 167,729 participants, LifeLines is one of the largest population based cohort studies of the world. A large amount of data is collected, biological samples are stored for future analyses (e.g., measurement of exposure biomarkers), and for a subsample of 15,638 participants genome wide genotype data are available. These numbers are large enough for studying effects of environmental exposures in vulnerable subgroups. This is important, because many factors (genetics, individual disease states, psychosocial stress, and socioeconomic status) have the potential to interact with environmental exposures [35]. The major strength of this study is the use of harmonized exposure models for ambient air pollution and for road traffic noise, and the use of validated questionnaires. Data harmonization facilitates comparability and combination with data from other cohorts and regions, which is beneficial for environmental epidemiology, where large sample sizes and broad exposure ranges are needed. Existing and future collaborations with other biobanks and international consortia hold the promise of answering complex questions in environmental epidemiology. Furthermore, the prospective nature of LifeLines allows research into long-term effects of environmental exposures. In addition, both objective (modeled) and subjective (questionnaire based) exposures were assessed. This enables studying effects of the exposure itself, and of perception of the exposure.

One limitation is that the results in this paper were based on the first 95,432 participants that were included in LifeLines. Geocoding and exposure estimation using the noise and air pollution models of the full cohort is currently ongoing. Since the inclusion of participants was independent of their place of residence, we have no reason to suspect geographical differences between participants in the first data release and the complete sample. Our study area is relatively rural compared to other parts of the Netherlands [36]. Levels of air pollution and noise exposures are lower than other parts of the Netherlands, due to for example lower population densities, and less extensive road networks. For example, exposure to NO_2_ in the EPIC-PROSPECT cohort located in the city of Utrecht and surrounding areas was on average 26.7 μg/m^3^ [37], compared to 15.7 μg/m^3^ in LifeLines. Conclusions based on research undertaken with LifeLines data will therefore be limited to exposure levels in that particular range. A major challenge in environmental epidemiology includes accurate exposure assessment [22, 38]. Actual measurement of individual-level exposures in a cohort as large as LifeLines would be impossible. Therefore other approaches to estimate exposures are used, such as land use regression modeling. Use of these models introduces misclassification of exposure to a varying degree; for example, due to daily mobility and long-term residential mobility [39]. In LifeLines, misclassification due to residential mobility can be tackled because data is available on address history, which is for some participants available from periods as early as year 1943. Future research should focus on characterizing exposures in earlier years, allowing for assessment of long-term exposures which is relevant for life course epidemiology. The collection of work addresses allows for outdoor exposure estimation of noise and air pollution at the work location. Combining exposures at home and work location will result in better estimation of an individual’s exposure [12].

## Conclusions

The combination of harmonized environmental exposures, relevant mediators and modifiers, and extensive assessment of multiple health outcomes makes LifeLines a great resource for environmental epidemiology, which it aims to be for the national and international scientific community. This paper provides an overview of the cohorts’ assessments that are relevant for environmental epidemiology. Key research questions that are investigated in LifeLines are about effects of noise, air pollution, and occupational exposures on healthy ageing [40–45].

## Abbreviations

BioSHaRE, Biobank Standardisation and Harmonisation for Research Excellence in the European Union; BMI, body mass index; CNOSSOS-EU, Common Noise Assessment Methods in Europe; CORINE, coordination of information on the environment; dB(A), Decibel (A); DBP, diastolic blood pressure; ECG, electrocardiogram; ESCAPE, European Study of Cohorts for Air Pollution Effects; EU-wide models, European wide models; FEV1, forced expiratory volume in 1 s; FVC, forced vital capacity; GIS, geographic information system; HYENA study, HYpertension and Exposure to Noise near Airports study; IQR, interquartile range; ISO, international Organization for Standardization; L_day_, a-weighted equivalent noise level over the 12-h day time period from 07.00 to 19.00 h; L_den_, a-weighted equivalent noise level over the day-evening-night time period of 24 h; L_night_, a-weighted equivalent noise level over the 8-h night time period from 23.00 to 07.00 h; LUR, land use regression; NO_2_ background, background level of nitrogen dioxide; NO_2_, nitrogen dioxide; PM_10_, particulate matter with diameter ≤10 μm; PM_2.5_ absorbance, reflectance on PM_2.5_ filters, i.e., marker of black carbon; PM_2.5_, particulate matter with diameter ≤2.5 μm; RMSE, root mean square error; SBP, systolic blood pressure; SD, Standard deviation; SOPs, standard operating procedures; WoON questionnaire, WoonOnderzoek Nederland
